# Bis(1-methyl­imidazole)[*meso*-α,α,α,α-tetra­kis(*o*-nicotinamido­phen­yl)porphinato]iron(II)–1-methyl­imidazole–tetra­hydro­furan (1/1/1.5)

**DOI:** 10.1107/S2414314621005319

**Published:** 2021-05-25

**Authors:** Yingying Fan, Jianfeng Li

**Affiliations:** a College of Materials Science and Optoelectronic Technology, CAS Center for Excellence in Topological Quantum Computation & Center of Materials Science and Optoelectronics Engineering, University of Chinese Academy of Sciences, Yanqi Lake, Huairou District, Beijing, 101408, People’s Republic of China; Benemérita Universidad Autónoma de Puebla, México

**Keywords:** crystal structure, 1-methyl­imidazole, porphyrin derivative, hydrogen bonds

## Abstract

In the title compound, [Fe^II^(C_68_H_44_N_12_O_4_)(C_4_H_6_N_2_)_2_]·C_4_H_6_N_2_·1.5C_4_H_8_O, the central Fe^II^ ion is coordinated by four pyrrole N atoms of the porphyrin core and two N atoms of the 1-methyl­imidazole ligands in the axial sites. One 1-methyl­imidazole and one and a half tetra­hydro­furan solvent mol­ecules are also present in the asymmetric unit. The complex exhibits a near planar porphyrin core conformation, in which the iron centre is slightly displaced towards the hindered porphyrin side (0.01 Å). The average Fe—N_p_ (N_p_ refers to the pyrrole nitro­gen atoms in the porphyrin) bond length is 1.990 (9) Å, and the axial Fe—N_Im_ (N_Im_ refers to the imidazole nitro­gen atoms) bond lengths are 1.993 (3) and 2.004 (3) Å. The dihedral angle between the two coordinated 1-methyl­imidazole planes is 56.6 (2)°. The dihedral angles between the 1-methyl­imidazole planes and the planes of the closest Fe—N_p_ vector are 16.8 (2) and 39.8 (2)°. N—H⋯N and N—H⋯O inter­actions are observed in the crystal structure.

## Abstract 2

The crystal structure of the bis­(1-methyl­imidazole)-ligated iron(II) picket-fence porphyrin derivative [Fe^II^(C_68_H_44_N_12_O_4_)(C_4_H_6_N_2_)_2_]·C_4_H_6_N_2_·1.5C_4_H_8_O is investigated.

## Graphical abstract

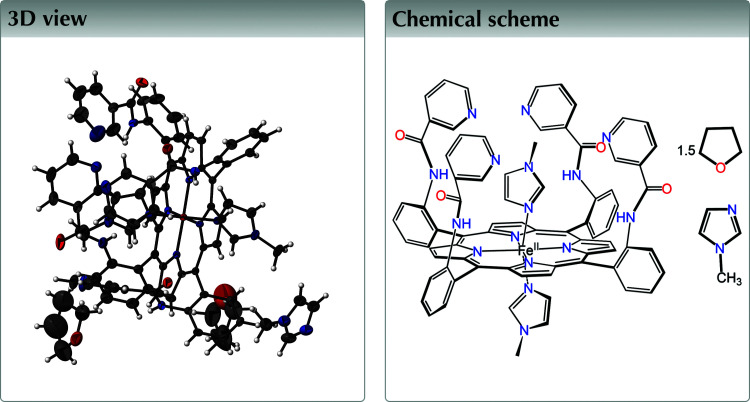

## Structure description

Heme *a* is an important redox site of cytochrome *c* oxidases (C*c*O) (Pitcher & Watmough, 2004 ▸), and bis­(imidazole)–iron(II) porphyrin complexes are used to understand the relationship between its structure and function (Walker, 2004 ▸). The picket-fence species with bis­(imidazole)-ligated groups is one of the effective models to study the effect of axial ligand orientation. Crystal structures of bis­(imidazole)-ligated iron(II) picket-fence porphyrinates, *e.g*. [Fe(TpivPP)(1-RIm)_2_] (TpivPP = *α,α,α,α*-tetra­kis­(*o*-pivalamido­phen­yl)porphyrin; 1-RIm = 1-methyl-, 1-ethyl-, or 1-vinyl­imidazole; Li *et al.*, 2008 ▸), [Fe(TImPP)(1-RIm)_2_] (TImPP = *α,α,α,α*-*o*-(1-methyl­imid­azole-5-carboxyl­amino­phen­yl)porphyrin; 1-RIm = 1-methyl- or 1-ethyl­imidazole; Yao *et al.*, 2017 ▸) and [Fe(MbenTpivPP)(1-MeIm)_2_] (MbenTpivPP = *meso*-mono[*α*-*o*-(benzene­carb­ox­amido)­phen­yl]tris­[*α,α,α*-*o*-(pivalamido­phen­yl)]porphyrin; 1-MeIm = 1-methyl­imidazole; He *et al.*, 2015 ▸) have been determined. Herein, the crystal structure of a new iron(II) porphyrin solvated complex, [Fe(C_68_H_44_N_12_O_4_)(C_4_H_6_N_2_)_2_]·C_4_H_6_N_2_·1.5C_4_H_8_O is reported.

**Figure 1:**
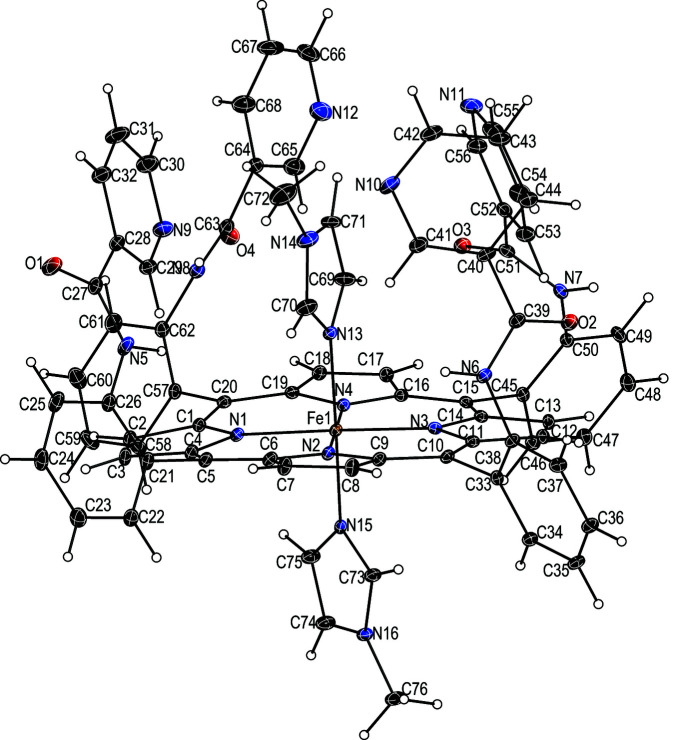
The mol­ecular entities in the title compound, with displacement ellipsoids drawn at the 25% probability level. The 1-methyl­imidazole and tetra­hydro­furan solvent mol­ecules are omitted for clarity.

**Figure 2:**
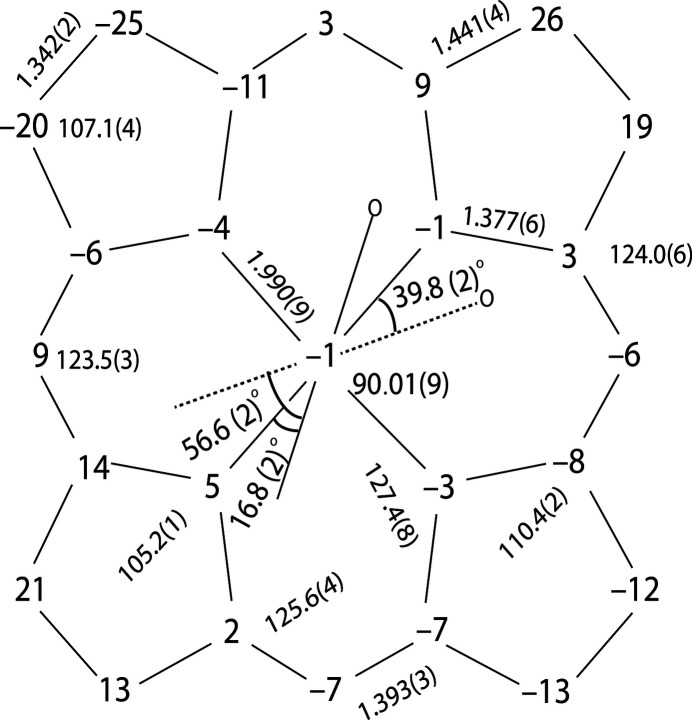
A formal diagram of the porphyrinato core of the title compound. Averaged values of the chemically unique bond distances (in Å) and angles (°) are shown. The numbers in parentheses are the e.s.d.’s calculated on the assumption that the averaged values were all drawn from the same population. The perpendicular displacements (in units of 0.01 Å) of the porphyrin core atoms from the 24-atom mean plane are also displayed. Positive values of the displacement are towards the hindered porphyrin side. The dashed line indicates the imidazole on the less hindered porphyrin side and the circles represent the positions of the methyl groups on the axial ligands.

The asymmetric unit of the title compound (Fig. 1 ▸) contains one bis­(1-methyl­imidazole)[*meso*-*α,α,α,α*-tetra­kis­(*o*-nicotin­am­ido­phen­yl)porphinato]iron(II), one 1-methyl­imidazole and one and a half tetra­hydro­furan lattice solvate mol­ecules. Additional qu­anti­tative information on the structure is given in Fig. 2 ▸, which displays the detailed displacement of each porphyrin core atom (in units of 0.01 Å) from the 24-atom mean plane. Averaged values of the chemically unique bond lengths (Å) and angles (°) are also shown. The title compound has a near planar porphyrin core conformation, in which the iron centre is slightly displaced towards the hindered porphyrin side (0.01 Å). The dihedral angles formed by the 1-MeIm axial ligand planes and the closest Fe—N_p_ vector are 16.8 (2) and 39.8 (2)°. The dihedral angle between the two coordinated imidazole planes is 56.6 (2)°, showing a relative perpendicular orientation. Fig. 2 ▸ also shows that the average N_p_—Fe—N_p_ angle is ideal at 90.01 (9)°, and the axial Fe—N_Im_ bond lengths are 1.993 (3) and 2.004 (3) Å. The average Fe—N_p_ distance of 1.990 (9) Å is a typical value for low-spin ferrous porphyrin derivatives (Scheidt & Reed, 1981 ▸).

**Figure 3:**
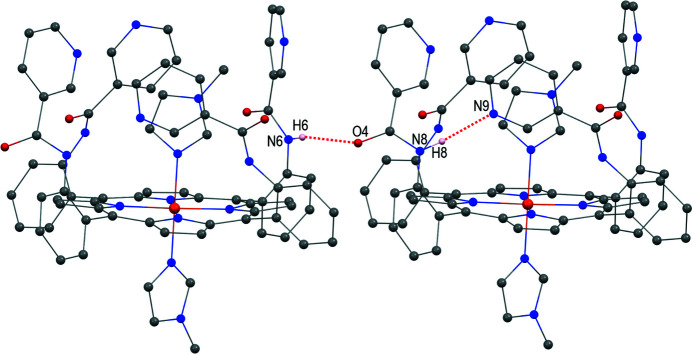
N—H⋯O and N—H⋯N inter­actions in the crystal structure of the title compound (see Table 1 ▸).

**Figure 4:**
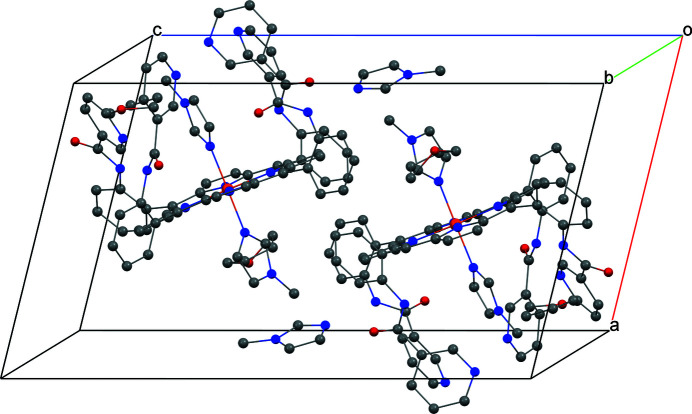
A view of the mol­ecular packing of the title compound. Hydrogen atoms are omitted for clarity.

Several intra- and inter-mol­ecular inter­actions are found in the title compound. As can be seen in Table 1 ▸ and Fig. 3 ▸, the distance between N8 and N9, and the N8—H8⋯N9 angle are 3.018 (5) Å and 156°, respectively, in agreement with reported values of 2.6 < N⋯N′ < 3.2 Å and 131.5 < N—H⋯N′ < 179.7° (Prasad & Govil, 1980 ▸; Aldilla *et al.*, 2017 ▸; Leigh *et al.*, 2013 ▸). The distance between N6 and O4, and the N6—H6⋯O4 angle are 2.948 (4) Å and 145°, respectively, consistent with the N—H⋯O inter­action of 2.7 < N⋯O < 3.05 Å and N—H⋯O > 130° (Bertolasi *et al.*, 1995 ▸; Malinovskii *et al.*, 2001 ▸). The packing structure (Fig. 4 ▸) shows that lattice solvent is placed in the voids left by the main mol­ecules in the crystal.

## Synthesis and crystallization

**General information.** All reactions and manipulations were carried out under argon using a double-manifold vacuum line and Schlenk wares. Tetra­hydro­furan (THF) was distilled from Na/benzo­phenone under N_2_. Hexanes were distilled over sodium/potassium alloy under N_2_. Solvents were degassed by repeated freeze–pump–thaw cycles. 1-MeIm was distilled under argon before use. Precursors H_2_TPyPP, [Fe(TPyPP)]Cl, and [Fe(TPyPP)]OH were prepared following literature methods (Gunter *et al.*, 1984 ▸; TPyPP is *o*-nicotinamido­phen­yl), with slight modifications.

**Synthesis of the title compound.** [Fe(TPyPP)]OH (10 mg, 8.6 × 10^−3^ mmol) and 1-MeIm (0.14 ml, 1.7 × 10 ^−3^ mol) were dissolved in 3 ml of THF. The mixture was stirred for 15 min and transferred into glass tubes (8 mm × 10 cm), which were layered with hexa­nes. Several days later, X-ray quality black block-shaped crystals were collected.

## Refinement

Crystal data, data collection and structure refinement details are summarized in Table 2 ▸. The atoms of THF mol­ecules (O5, C77, C78, C79, C80 and O6, C81, C82, C83, C84) exhibited unusual thermal motions and were thus restrained using the RIGU, ISOR and DFIX commands (Sheldrick, 2015*b* ▸). The O6⋯C84 THF mol­ecule was refined with a fixed occupancy of 1/2. Seven outlier reflections were omitted in the last cycles of refinement.

## Supplementary Material

Crystal structure: contains datablock(s) I. DOI: 10.1107/S2414314621005319/bh4061sup1.cif

Structure factors: contains datablock(s) I. DOI: 10.1107/S2414314621005319/bh4061Isup2.hkl

CCDC reference: 2068473

Additional supporting information:  crystallographic information; 3D view; checkCIF report

## Figures and Tables

**Table 1:** Hydrogen-bond geometry (Å, °)

*D*—H⋯*A*	*D*—H	H⋯*A*	*D*⋯*A*	*D*—H⋯*A*
N6—H6⋯O4^i^	0.88	2.18	2.948 (4)	145
N8—H8⋯N9	0.88	2.19	3.018 (5)	156

Symmetry code: (i) 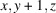 .

**Table 2:** Experimental details

Crystal data	
Chemical formula	[Fe(C_68_H_44_N_12_O_4_)(C_4_H_6_N_2_)_2_]·C_4_H_6_N_2_·1.5C_4_H_8_O
*M*_r_	1503.48
Crystal system, space group	Triclinic, *P* 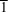
Temperature (K)	100
*a*, *b*, *c* (Å)	13.0880 (18), 13.8413 (18), 22.771 (3)
α, β, γ (°)	75.588 (5), 76.138 (4), 74.316 (4)
*V* (Å^3^)	3780.3 (9)
*Z*	2
Radiation type	Mo *K*α
μ (mm^−1^)	0.27
Crystal size (mm)	0.55 × 0.16 × 0.07

Data collection	
Diffractometer	Bruker D8 QUEST System
Absorption correction	Multi-scan (*SADABS*; Bruker, 2016 ▸)
*T*_min_, *T*_max_	0.950, 0.981
No. of measured, independent and observed [*I* > 2σ(*I*)] reflections	67154, 16109, 11563
*R*_int_	0.069
(sin θ/λ)_max_ (Å^−1^)	0.634

Refinement	
*R*[*F* ^2^ > 2σ(*F* ^2^)], *wR*(*F* ^2^), *S*	0.070, 0.219, 1.03
No. of reflections	16109
No. of parameters	1022
No. of restraints	131
H-atom treatment	H-atom parameters constrained
Δρ_max_, Δρ_min_ (e Å^−3^)	1.82, −0.80

Computer programs: *APEX2* and *SAINT* (Bruker, 2013 ▸), *SHELXT2014/6* (Sheldrick, 2015*a* ▸), *SHELXL2014/6* (Sheldrick, 2015*b* ▸), *Mercury* (Macrae *et al.*, 2020 ▸) and *enCIFer* (Allen *et al.*, 2004 ▸).

## full crystallographic data

### Crystal data

**Table:**

[Fe(C_68_H_44_N_12_O_4_)(C_4_H_6_N_2_)_2_]·C_4_H_6_N_2_·1.5C_4_H_8_O	*Z* = 2
*M**_r_* = 1503.48	*F*(000) = 1572
Triclinic, *P*1	*D*_x_ = 1.321 Mg m^−^^3^
*a* = 13.0880 (18) Å	Mo *K*α radiation, λ = 0.71073 Å
*b* = 13.8413 (18) Å	Cell parameters from 9990 reflections
*c* = 22.771 (3) Å	θ = 2.3–26.6°
α = 75.588 (5)°	µ = 0.27 mm^−^^1^
β = 76.138 (4)°	*T* = 100 K
γ = 74.316 (4)°	Block, black
*V* = 3780.3 (9) Å^3^	0.55 × 0.16 × 0.07 mm

### Data collection

**Table:**

Bruker D8 QUEST System diffractometer	11563 reflections with *I* > 2σ(*I*)
Radiation source: fine-focus sealed tube	*R*_int_ = 0.069
φ and ω scans	θ_max_ = 26.8°, θ_min_ = 2.2°
Absorption correction: multi-scan (SADABS; Bruker, 2016)	*h* = −16→16
*T*_min_ = 0.950, *T*_max_ = 0.981	*k* = −17→17
67154 measured reflections	*l* = −28→28
16109 independent reflections	

### Refinement

**Table:**

Refinement on *F*^2^	Hydrogen site location: inferred from neighbouring sites
Least-squares matrix: full	H-atom parameters constrained
*R*[*F*^2^ > 2σ(*F*^2^)] = 0.070	*w* = 1/[σ^2^(*F*_o_^2^) + (0.1118*P*)^2^ + 7.2076*P*] where *P* = (*F*_o_^2^ + 2*F*_c_^2^)/3
*wR*(*F*^2^) = 0.219	(Δ/σ)_max_ = 0.001
*S* = 1.03	Δρ_max_ = 1.82 e Å^−^^3^
16109 reflections	Δρ_min_ = −0.80 e Å^−^^3^
1022 parameters	Extinction correction: SHELXL2016/6 (Sheldrick 2015b), Fc^*^=kFc[1+0.001xFc^2^λ^3^/sin(2θ)]^-1/4^
131 restraints	Extinction coefficient: 0.0029 (8)

### Fractional atomic coordinates and isotropic or equivalent isotropic displacement parameters (Å^2^)

**Table:**

	*x*	*y*	*z*	*U*_iso_*/*U*_eq_	Occ. (<1)
Fe1	0.58466 (3)	0.34410 (3)	0.29656 (2)	0.01633 (14)	
O1	0.7193 (3)	0.5115 (3)	−0.03486 (13)	0.0573 (9)	
O2	0.9173 (2)	0.5353 (2)	0.37668 (12)	0.0344 (6)	
O3	0.8951 (2)	0.0304 (2)	0.35901 (12)	0.0347 (6)	
O4	0.7501 (2)	−0.1694 (2)	0.21998 (13)	0.0394 (7)	
N1	0.5234 (2)	0.3438 (2)	0.22501 (12)	0.0194 (5)	
N2	0.5694 (2)	0.4958 (2)	0.27220 (12)	0.0187 (5)	
N3	0.6423 (2)	0.34404 (19)	0.36965 (12)	0.0166 (5)	
N4	0.6033 (2)	0.19258 (19)	0.31977 (12)	0.0177 (5)	
N5	0.6301 (3)	0.4986 (2)	0.06434 (14)	0.0350 (7)	
H5	0.635418	0.461177	0.101351	0.042*	
N6	0.8048 (2)	0.6503 (2)	0.31729 (13)	0.0238 (6)	
H6	0.795126	0.682020	0.279757	0.029*	
N7	0.8873 (2)	0.0955 (2)	0.44292 (13)	0.0242 (6)	
H7	0.917595	0.130964	0.458131	0.029*	
N8	0.7052 (2)	0.0007 (2)	0.17919 (13)	0.0262 (6)	
H8	0.728899	0.057460	0.165227	0.031*	
N9	0.7835 (3)	0.1828 (3)	0.09547 (17)	0.0411 (8)	
N10	1.0454 (3)	0.5801 (4)	0.16177 (17)	0.0543 (10)	
N11	1.1474 (3)	0.1740 (3)	0.26412 (18)	0.0489 (9)	
N12	1.0272 (4)	0.0024 (4)	0.1888 (2)	0.0707 (14)	
N13	0.7336 (2)	0.3273 (2)	0.24526 (12)	0.0219 (6)	
N14	0.8756 (3)	0.3565 (3)	0.17385 (17)	0.0467 (9)	
N15	0.4359 (2)	0.3608 (2)	0.34675 (12)	0.0185 (5)	
N16	0.2869 (2)	0.4160 (2)	0.40966 (13)	0.0266 (6)	
C1	0.5042 (3)	0.2616 (2)	0.20833 (14)	0.0205 (6)	
C2	0.4499 (3)	0.2975 (3)	0.15633 (16)	0.0265 (7)	
H2	0.427468	0.256355	0.136304	0.032*	
C3	0.4368 (3)	0.3998 (3)	0.14125 (16)	0.0276 (7)	
H3	0.402477	0.444495	0.109128	0.033*	
C4	0.4847 (3)	0.4285 (2)	0.18296 (15)	0.0229 (7)	
C5	0.4974 (3)	0.5279 (3)	0.17715 (14)	0.0230 (7)	
C6	0.5386 (3)	0.5577 (2)	0.21915 (15)	0.0225 (7)	
C7	0.5532 (3)	0.6593 (3)	0.21309 (16)	0.0291 (8)	
H7A	0.540834	0.714970	0.179530	0.035*	
C8	0.5878 (3)	0.6607 (3)	0.26386 (16)	0.0281 (7)	
H8A	0.602251	0.717902	0.273650	0.034*	
C9	0.5987 (3)	0.5586 (2)	0.30055 (14)	0.0196 (6)	
C10	0.6347 (2)	0.5280 (2)	0.35650 (14)	0.0188 (6)	
C11	0.6556 (2)	0.4266 (2)	0.38797 (14)	0.0182 (6)	
C12	0.6964 (3)	0.3938 (2)	0.44465 (15)	0.0215 (7)	
H12	0.713714	0.436296	0.466068	0.026*	
C13	0.7057 (3)	0.2925 (2)	0.46181 (15)	0.0210 (6)	
H13	0.729531	0.249690	0.497841	0.025*	
C14	0.6722 (2)	0.2609 (2)	0.41477 (14)	0.0174 (6)	
C15	0.6697 (2)	0.1605 (2)	0.41657 (14)	0.0167 (6)	
C16	0.6398 (2)	0.1296 (2)	0.37062 (14)	0.0178 (6)	
C17	0.6468 (3)	0.0238 (2)	0.37023 (15)	0.0223 (7)	
H17	0.670880	−0.033983	0.400284	0.027*	
C18	0.6127 (3)	0.0226 (2)	0.31940 (15)	0.0223 (7)	
H18	0.608180	−0.036083	0.306560	0.027*	
C19	0.5842 (2)	0.1280 (2)	0.28786 (14)	0.0189 (6)	
C20	0.5373 (2)	0.1593 (2)	0.23558 (15)	0.0199 (6)	
C21	0.4763 (3)	0.6027 (3)	0.11916 (15)	0.0260 (7)	
C22	0.3913 (3)	0.6880 (3)	0.11910 (17)	0.0315 (8)	
H22	0.343359	0.698150	0.156734	0.038*	
C23	0.3747 (4)	0.7590 (3)	0.06523 (19)	0.0412 (10)	
H23	0.316259	0.817299	0.065979	0.049*	
C24	0.4445 (4)	0.7437 (3)	0.01048 (19)	0.0432 (10)	
H24	0.433220	0.791659	−0.026580	0.052*	
C25	0.5305 (4)	0.6594 (3)	0.00883 (18)	0.0400 (10)	
H25	0.578584	0.650280	−0.028929	0.048*	
C26	0.5459 (3)	0.5882 (3)	0.06286 (16)	0.0310 (8)	
C27	0.7031 (3)	0.4618 (3)	0.01773 (16)	0.0351 (9)	
C28	0.7651 (3)	0.3536 (3)	0.03375 (17)	0.0331 (8)	
C29	0.7316 (3)	0.2810 (3)	0.08406 (17)	0.0327 (8)	
H29	0.667679	0.302707	0.112130	0.039*	
C30	0.8741 (4)	0.1540 (4)	0.0558 (2)	0.0518 (12)	
H30	0.912181	0.084487	0.063007	0.062*	
C31	0.9149 (4)	0.2203 (4)	0.0050 (2)	0.0603 (14)	
H31	0.980064	0.196685	−0.021651	0.072*	
C32	0.8601 (4)	0.3211 (4)	−0.00666 (19)	0.0463 (11)	
H32	0.886514	0.367822	−0.041589	0.056*	
C33	0.6418 (3)	0.6083 (2)	0.38832 (14)	0.0190 (6)	
C34	0.5649 (3)	0.6259 (3)	0.44132 (15)	0.0234 (7)	
H34	0.511034	0.586948	0.455665	0.028*	
C35	0.5653 (3)	0.6987 (3)	0.47335 (16)	0.0276 (7)	
H35	0.512816	0.708596	0.509480	0.033*	
C36	0.6418 (3)	0.7564 (3)	0.45278 (17)	0.0295 (8)	
H36	0.642218	0.806727	0.474467	0.035*	
C37	0.7181 (3)	0.7412 (3)	0.40050 (17)	0.0271 (7)	
H37	0.770327	0.782001	0.385906	0.033*	
C38	0.7195 (3)	0.6666 (2)	0.36884 (15)	0.0212 (7)	
C39	0.9001 (3)	0.5861 (3)	0.32628 (16)	0.0248 (7)	
C40	0.9877 (3)	0.5806 (3)	0.27001 (16)	0.0290 (8)	
C41	0.9675 (3)	0.5903 (4)	0.21235 (19)	0.0412 (10)	
H41	0.894341	0.605087	0.207576	0.049*	
C42	1.1475 (4)	0.5602 (4)	0.1702 (2)	0.0503 (12)	
H42	1.203890	0.553055	0.135416	0.060*	
C43	1.1749 (3)	0.5496 (3)	0.2261 (2)	0.0433 (10)	
H43	1.248605	0.535337	0.229657	0.052*	
C44	1.0942 (3)	0.5598 (3)	0.27734 (19)	0.0345 (8)	
H44	1.111231	0.552825	0.316683	0.041*	
C45	0.6993 (3)	0.0804 (2)	0.47120 (15)	0.0194 (6)	
C46	0.6203 (3)	0.0351 (3)	0.51272 (16)	0.0249 (7)	
H46	0.547963	0.056304	0.506011	0.030*	
C47	0.6446 (3)	−0.0399 (3)	0.56352 (17)	0.0301 (8)	
H47	0.589642	−0.070265	0.590995	0.036*	
C48	0.7502 (3)	−0.0705 (3)	0.57402 (17)	0.0337 (8)	
H48	0.767784	−0.121652	0.608846	0.040*	
C49	0.8292 (3)	−0.0259 (3)	0.53334 (17)	0.0295 (8)	
H49	0.901284	−0.046803	0.540392	0.035*	
C50	0.8047 (3)	0.0487 (2)	0.48251 (15)	0.0220 (7)	
C51	0.9211 (3)	0.0880 (3)	0.38313 (16)	0.0271 (7)	
C52	0.9964 (3)	0.1547 (3)	0.34652 (16)	0.0281 (7)	
C53	0.9861 (3)	0.2524 (3)	0.35526 (19)	0.0372 (9)	
H53	0.930751	0.279764	0.386133	0.045*	
C54	1.0574 (4)	0.3092 (3)	0.3185 (2)	0.0468 (11)	
H54	1.052342	0.376491	0.323615	0.056*	
C55	1.1358 (4)	0.2677 (4)	0.2743 (2)	0.0497 (11)	
H55	1.184956	0.307713	0.249515	0.060*	
C56	1.0774 (3)	0.1195 (3)	0.3000 (2)	0.0394 (9)	
H56	1.083488	0.052972	0.293350	0.047*	
C57	0.5139 (3)	0.0806 (3)	0.20926 (15)	0.0227 (7)	
C58	0.4059 (3)	0.0830 (3)	0.20982 (18)	0.0323 (8)	
H58	0.350184	0.131934	0.228657	0.039*	
C59	0.3788 (4)	0.0159 (3)	0.1837 (2)	0.0454 (11)	
H59	0.305090	0.019662	0.184008	0.054*	
C60	0.4582 (4)	−0.0563 (3)	0.1571 (2)	0.0491 (12)	
H60	0.439354	−0.102509	0.139085	0.059*	
C61	0.5651 (3)	−0.0620 (3)	0.1565 (2)	0.0395 (9)	
H61	0.619824	−0.112371	0.138269	0.047*	
C62	0.5932 (3)	0.0056 (3)	0.18252 (16)	0.0261 (7)	
C63	0.7756 (3)	−0.0872 (3)	0.19658 (16)	0.0278 (7)	
C64	0.8912 (3)	−0.0806 (3)	0.18496 (17)	0.0305 (8)	
C65	0.9233 (4)	−0.0037 (4)	0.1989 (3)	0.0552 (13)	
H65	0.869474	0.047861	0.216560	0.066*	
C66	1.1009 (4)	−0.0725 (5)	0.1643 (3)	0.0619 (14)	
H66	1.175063	−0.071413	0.158030	0.074*	
C67	1.0758 (4)	−0.1478 (5)	0.1485 (3)	0.0658 (15)	
H67	1.131010	−0.197243	0.129846	0.079*	
C68	0.9698 (4)	−0.1544 (4)	0.1591 (2)	0.0540 (12)	
H68	0.951208	−0.209012	0.148768	0.065*	
C69	0.8132 (3)	0.2408 (3)	0.2457 (2)	0.0403 (10)	
H69	0.808780	0.176830	0.272555	0.048*	
C70	0.7735 (3)	0.3964 (3)	0.2004 (2)	0.0417 (10)	
H70	0.735439	0.464950	0.188377	0.050*	
C71	0.9001 (3)	0.2596 (4)	0.20197 (19)	0.0398 (10)	
H71	0.966379	0.211919	0.193141	0.048*	
C72	0.9429 (5)	0.4088 (5)	0.1217 (3)	0.0746 (18)	
H72A	1.009003	0.410523	0.134106	0.112*	
H72B	0.903172	0.478948	0.108413	0.112*	
H72C	0.961726	0.372137	0.087557	0.112*	
C73	0.3874 (3)	0.4273 (3)	0.38259 (16)	0.0276 (7)	
H73	0.419327	0.477204	0.388636	0.033*	
C74	0.2703 (3)	0.3381 (3)	0.39007 (19)	0.0349 (9)	
H74	0.206625	0.312165	0.401208	0.042*	
C75	0.3623 (3)	0.3042 (3)	0.35137 (19)	0.0345 (9)	
H75	0.373814	0.249436	0.330618	0.041*	
C76	0.2109 (3)	0.4733 (3)	0.4539 (2)	0.0395 (10)	
H76A	0.218505	0.436576	0.495892	0.059*	
H76B	0.137079	0.480815	0.448195	0.059*	
H76C	0.226070	0.541211	0.447238	0.059*	
O5	0.2857 (6)	0.6768 (6)	0.3207 (3)	0.156 (3)	
C77	0.3330 (7)	0.8184 (6)	0.3382 (4)	0.127 (3)	
H77A	0.405675	0.825530	0.314817	0.152*	
H77B	0.311978	0.857899	0.371626	0.152*	
C78	0.3289 (5)	0.7083 (6)	0.3629 (3)	0.089 (2)	
H78A	0.402203	0.666084	0.366424	0.107*	
H78B	0.282335	0.700512	0.404258	0.107*	
C79	0.2486 (7)	0.8521 (7)	0.2954 (4)	0.114 (3)	
H79A	0.174822	0.874122	0.318532	0.137*	
H79B	0.265453	0.908634	0.260950	0.137*	
C80	0.2597 (6)	0.7592 (7)	0.2731 (4)	0.118 (3)	
H80A	0.191263	0.758752	0.261946	0.141*	
H80B	0.317519	0.755171	0.236083	0.141*	
O6	0.8138 (5)	0.8462 (5)	0.0574 (3)	0.0590 (17)	0.5
C81	0.7780 (13)	0.9096 (11)	0.0030 (6)	0.106 (4)	0.5
H81A	0.833178	0.946314	−0.023455	0.127*	0.5
H81B	0.709378	0.959525	0.012889	0.127*	0.5
C82	0.7627 (19)	0.8267 (13)	−0.0271 (10)	0.151 (6)	0.5
H82A	0.685374	0.839013	−0.029143	0.181*	0.5
H82B	0.803946	0.833595	−0.069877	0.181*	0.5
C83	0.7993 (14)	0.7171 (13)	0.0077 (7)	0.116 (5)	0.5
H83A	0.747106	0.674427	0.012148	0.139*	0.5
H83B	0.872100	0.682948	−0.011347	0.139*	0.5
C84	0.7989 (10)	0.7451 (8)	0.0686 (6)	0.077 (3)	0.5
H84A	0.729297	0.740092	0.096877	0.093*	0.5
H84B	0.857536	0.696697	0.088429	0.093*	0.5
N17	0.0059 (3)	0.7796 (3)	0.40172 (16)	0.0375 (8)	
N18	0.0525 (3)	0.7857 (3)	0.48807 (17)	0.0389 (8)	
C85	0.0185 (3)	0.6969 (3)	0.4962 (2)	0.0410 (9)	
H85	0.015528	0.646219	0.532983	0.049*	
C86	0.0447 (4)	0.8320 (3)	0.4308 (2)	0.0440 (10)	
H86	0.064341	0.895436	0.411955	0.053*	
C87	−0.0108 (3)	0.6926 (3)	0.4428 (2)	0.0406 (9)	
H87	−0.037361	0.639611	0.436074	0.049*	
C88	−0.0119 (4)	0.8112 (4)	0.3383 (2)	0.0458 (10)	
H88A	0.012233	0.874904	0.319161	0.069*	
H88B	−0.088957	0.822319	0.338055	0.069*	
H88C	0.029166	0.757643	0.315163	0.069*	

### Atomic displacement parameters (Å^2^)

**Table:**

	*U*^11^	*U*^22^	*U*^33^	*U*^12^	*U*^13^	*U*^23^
Fe1	0.0180 (2)	0.0162 (2)	0.0160 (2)	−0.00469 (17)	−0.00201 (17)	−0.00551 (16)
O1	0.056 (2)	0.069 (2)	0.0226 (15)	0.0063 (17)	0.0020 (13)	0.0037 (14)
O2	0.0355 (14)	0.0349 (14)	0.0275 (14)	−0.0039 (11)	−0.0031 (11)	−0.0037 (11)
O3	0.0314 (14)	0.0386 (15)	0.0373 (15)	−0.0101 (12)	−0.0031 (11)	−0.0139 (12)
O4	0.0412 (16)	0.0273 (14)	0.0465 (17)	−0.0083 (12)	−0.0096 (13)	0.0002 (12)
N1	0.0220 (13)	0.0189 (13)	0.0168 (13)	−0.0050 (11)	−0.0011 (10)	−0.0049 (10)
N2	0.0203 (13)	0.0206 (13)	0.0164 (13)	−0.0064 (11)	−0.0020 (10)	−0.0053 (10)
N3	0.0159 (12)	0.0136 (12)	0.0200 (13)	−0.0032 (10)	−0.0023 (10)	−0.0041 (10)
N4	0.0173 (12)	0.0185 (13)	0.0187 (13)	−0.0037 (10)	−0.0034 (10)	−0.0067 (10)
N5	0.048 (2)	0.0327 (17)	0.0169 (14)	−0.0007 (15)	−0.0039 (13)	−0.0024 (12)
N6	0.0261 (15)	0.0251 (14)	0.0198 (14)	−0.0081 (12)	0.0010 (11)	−0.0061 (11)
N7	0.0206 (14)	0.0259 (15)	0.0279 (15)	−0.0064 (11)	−0.0076 (11)	−0.0047 (12)
N8	0.0301 (15)	0.0219 (14)	0.0279 (15)	−0.0057 (12)	−0.0035 (12)	−0.0095 (12)
N9	0.0373 (19)	0.0370 (19)	0.044 (2)	−0.0062 (15)	0.0024 (15)	−0.0121 (16)
N10	0.048 (2)	0.080 (3)	0.0317 (19)	−0.013 (2)	0.0092 (16)	−0.0215 (19)
N11	0.0282 (18)	0.061 (3)	0.051 (2)	−0.0142 (17)	0.0067 (16)	−0.0089 (19)
N12	0.052 (3)	0.093 (4)	0.088 (3)	−0.024 (2)	−0.010 (2)	−0.050 (3)
N13	0.0223 (14)	0.0247 (14)	0.0199 (13)	−0.0063 (11)	−0.0009 (11)	−0.0082 (11)
N14	0.0368 (19)	0.057 (2)	0.046 (2)	−0.0167 (17)	0.0125 (16)	−0.0212 (18)
N15	0.0214 (13)	0.0184 (13)	0.0162 (13)	−0.0035 (10)	−0.0049 (10)	−0.0039 (10)
N16	0.0201 (14)	0.0323 (16)	0.0285 (15)	−0.0046 (12)	0.0004 (12)	−0.0140 (13)
C1	0.0203 (15)	0.0234 (16)	0.0201 (16)	−0.0045 (13)	−0.0038 (12)	−0.0085 (13)
C2	0.0308 (18)	0.0295 (18)	0.0227 (17)	−0.0059 (14)	−0.0079 (14)	−0.0098 (14)
C3	0.0357 (19)	0.0287 (18)	0.0206 (17)	−0.0046 (15)	−0.0111 (14)	−0.0063 (14)
C4	0.0277 (17)	0.0220 (16)	0.0187 (15)	−0.0021 (13)	−0.0054 (13)	−0.0063 (13)
C5	0.0273 (17)	0.0231 (16)	0.0166 (15)	−0.0024 (13)	−0.0036 (13)	−0.0041 (12)
C6	0.0273 (17)	0.0200 (16)	0.0190 (15)	−0.0052 (13)	−0.0008 (13)	−0.0054 (12)
C7	0.043 (2)	0.0224 (17)	0.0224 (17)	−0.0102 (15)	−0.0090 (15)	0.0013 (13)
C8	0.039 (2)	0.0216 (17)	0.0270 (18)	−0.0108 (15)	−0.0099 (15)	−0.0026 (14)
C9	0.0229 (16)	0.0168 (15)	0.0198 (15)	−0.0055 (12)	−0.0026 (12)	−0.0051 (12)
C10	0.0198 (15)	0.0201 (15)	0.0184 (15)	−0.0070 (12)	−0.0003 (12)	−0.0075 (12)
C11	0.0160 (14)	0.0192 (15)	0.0212 (15)	−0.0052 (12)	−0.0013 (12)	−0.0082 (12)
C12	0.0224 (16)	0.0213 (16)	0.0248 (16)	−0.0057 (13)	−0.0066 (13)	−0.0087 (13)
C13	0.0234 (16)	0.0228 (16)	0.0190 (15)	−0.0042 (13)	−0.0084 (12)	−0.0050 (12)
C14	0.0143 (14)	0.0182 (15)	0.0210 (15)	−0.0045 (12)	−0.0028 (12)	−0.0059 (12)
C15	0.0131 (14)	0.0178 (15)	0.0200 (15)	−0.0040 (11)	−0.0028 (11)	−0.0047 (12)
C16	0.0169 (14)	0.0174 (15)	0.0190 (15)	−0.0037 (12)	−0.0021 (12)	−0.0049 (12)
C17	0.0254 (17)	0.0179 (15)	0.0244 (16)	−0.0050 (13)	−0.0059 (13)	−0.0045 (12)
C18	0.0262 (17)	0.0173 (15)	0.0245 (17)	−0.0046 (13)	−0.0047 (13)	−0.0068 (13)
C19	0.0187 (15)	0.0176 (15)	0.0224 (16)	−0.0060 (12)	−0.0020 (12)	−0.0072 (12)
C20	0.0179 (15)	0.0224 (16)	0.0218 (16)	−0.0063 (12)	−0.0009 (12)	−0.0094 (13)
C21	0.0359 (19)	0.0244 (17)	0.0192 (16)	−0.0068 (15)	−0.0089 (14)	−0.0031 (13)
C22	0.038 (2)	0.0289 (19)	0.0282 (19)	−0.0054 (16)	−0.0089 (16)	−0.0062 (15)
C23	0.050 (3)	0.031 (2)	0.040 (2)	−0.0009 (18)	−0.0170 (19)	−0.0036 (17)
C24	0.059 (3)	0.041 (2)	0.028 (2)	−0.007 (2)	−0.0197 (19)	0.0025 (17)
C25	0.057 (3)	0.037 (2)	0.0214 (18)	−0.0043 (19)	−0.0091 (17)	−0.0032 (16)
C26	0.040 (2)	0.0295 (19)	0.0235 (18)	−0.0046 (16)	−0.0102 (15)	−0.0053 (14)
C27	0.035 (2)	0.047 (2)	0.0195 (18)	−0.0057 (17)	−0.0052 (15)	−0.0032 (16)
C28	0.0316 (19)	0.043 (2)	0.0243 (18)	−0.0055 (17)	−0.0026 (15)	−0.0110 (16)
C29	0.0290 (19)	0.036 (2)	0.032 (2)	−0.0069 (16)	0.0004 (15)	−0.0109 (16)
C30	0.049 (3)	0.043 (3)	0.051 (3)	0.004 (2)	0.000 (2)	−0.011 (2)
C31	0.048 (3)	0.063 (3)	0.045 (3)	0.011 (2)	0.013 (2)	−0.011 (2)
C32	0.040 (2)	0.056 (3)	0.031 (2)	−0.003 (2)	0.0031 (18)	−0.0045 (19)
C33	0.0214 (15)	0.0158 (14)	0.0212 (16)	−0.0038 (12)	−0.0069 (12)	−0.0041 (12)
C34	0.0232 (16)	0.0224 (16)	0.0249 (17)	−0.0083 (13)	−0.0003 (13)	−0.0056 (13)
C35	0.0278 (18)	0.0290 (18)	0.0246 (17)	−0.0047 (14)	0.0038 (14)	−0.0129 (14)
C36	0.035 (2)	0.0229 (17)	0.0340 (19)	−0.0062 (15)	−0.0021 (15)	−0.0166 (15)
C37	0.0279 (18)	0.0213 (17)	0.0357 (19)	−0.0131 (14)	0.0000 (15)	−0.0092 (14)
C38	0.0251 (16)	0.0180 (15)	0.0202 (16)	−0.0059 (13)	−0.0008 (13)	−0.0052 (12)
C39	0.0284 (18)	0.0213 (16)	0.0263 (18)	−0.0085 (14)	0.0001 (14)	−0.0089 (14)
C40	0.0317 (19)	0.0251 (17)	0.0274 (18)	−0.0087 (15)	0.0070 (15)	−0.0094 (14)
C41	0.033 (2)	0.056 (3)	0.033 (2)	−0.0119 (19)	0.0047 (17)	−0.0149 (19)
C42	0.040 (2)	0.062 (3)	0.042 (2)	−0.011 (2)	0.0138 (19)	−0.019 (2)
C43	0.032 (2)	0.044 (2)	0.051 (3)	−0.0132 (18)	0.0075 (18)	−0.013 (2)
C44	0.032 (2)	0.033 (2)	0.038 (2)	−0.0111 (16)	0.0014 (16)	−0.0095 (16)
C45	0.0214 (15)	0.0160 (15)	0.0233 (16)	−0.0031 (12)	−0.0068 (13)	−0.0071 (12)
C46	0.0234 (16)	0.0246 (17)	0.0280 (18)	−0.0060 (13)	−0.0053 (14)	−0.0064 (14)
C47	0.0317 (19)	0.0297 (19)	0.0283 (18)	−0.0110 (15)	−0.0055 (15)	−0.0002 (15)
C48	0.039 (2)	0.0307 (19)	0.0286 (19)	−0.0079 (16)	−0.0127 (16)	0.0057 (15)
C49	0.0234 (17)	0.0313 (19)	0.0326 (19)	−0.0005 (14)	−0.0137 (15)	−0.0020 (15)
C50	0.0222 (16)	0.0215 (16)	0.0241 (16)	−0.0033 (13)	−0.0067 (13)	−0.0072 (13)
C51	0.0187 (16)	0.0289 (18)	0.0311 (19)	0.0008 (14)	−0.0064 (14)	−0.0058 (15)
C52	0.0217 (17)	0.0317 (19)	0.0294 (18)	−0.0045 (14)	−0.0076 (14)	−0.0021 (15)
C53	0.034 (2)	0.032 (2)	0.039 (2)	−0.0035 (16)	−0.0032 (17)	−0.0026 (17)
C54	0.046 (3)	0.033 (2)	0.057 (3)	−0.0117 (19)	−0.008 (2)	−0.001 (2)
C55	0.037 (2)	0.052 (3)	0.057 (3)	−0.020 (2)	−0.006 (2)	0.004 (2)
C56	0.028 (2)	0.043 (2)	0.046 (2)	−0.0094 (17)	0.0012 (17)	−0.0118 (19)
C57	0.0264 (17)	0.0235 (16)	0.0219 (16)	−0.0067 (13)	−0.0072 (13)	−0.0074 (13)
C58	0.0279 (19)	0.0314 (19)	0.043 (2)	−0.0070 (15)	−0.0106 (16)	−0.0135 (17)
C59	0.038 (2)	0.043 (2)	0.069 (3)	−0.0109 (19)	−0.023 (2)	−0.022 (2)
C60	0.054 (3)	0.040 (2)	0.072 (3)	−0.012 (2)	−0.028 (2)	−0.027 (2)
C61	0.045 (2)	0.032 (2)	0.049 (2)	−0.0039 (17)	−0.0126 (19)	−0.0224 (18)
C62	0.0293 (18)	0.0236 (17)	0.0278 (18)	−0.0047 (14)	−0.0076 (14)	−0.0085 (14)
C63	0.0326 (19)	0.0271 (18)	0.0237 (17)	−0.0046 (15)	−0.0040 (14)	−0.0082 (14)
C64	0.0304 (19)	0.0310 (19)	0.0308 (19)	−0.0038 (15)	−0.0060 (15)	−0.0106 (15)
C65	0.040 (2)	0.064 (3)	0.072 (3)	−0.011 (2)	−0.006 (2)	−0.039 (3)
C66	0.030 (2)	0.082 (4)	0.080 (4)	−0.012 (2)	−0.009 (2)	−0.029 (3)
C67	0.031 (2)	0.077 (4)	0.089 (4)	−0.008 (2)	0.011 (2)	−0.043 (3)
C68	0.042 (3)	0.053 (3)	0.070 (3)	−0.013 (2)	0.002 (2)	−0.025 (2)
C69	0.028 (2)	0.037 (2)	0.048 (2)	0.0004 (17)	0.0011 (17)	−0.0123 (18)
C70	0.040 (2)	0.039 (2)	0.043 (2)	−0.0125 (18)	0.0078 (18)	−0.0118 (18)
C71	0.0188 (18)	0.055 (3)	0.043 (2)	−0.0009 (17)	0.0038 (16)	−0.022 (2)
C72	0.067 (4)	0.088 (4)	0.070 (4)	−0.040 (3)	0.025 (3)	−0.028 (3)
C73	0.0258 (17)	0.0298 (18)	0.0315 (19)	−0.0096 (14)	0.0007 (14)	−0.0155 (15)
C74	0.0251 (18)	0.042 (2)	0.044 (2)	−0.0172 (16)	0.0057 (16)	−0.0208 (18)
C75	0.0289 (19)	0.039 (2)	0.043 (2)	−0.0168 (16)	0.0044 (16)	−0.0226 (18)
C76	0.0265 (19)	0.050 (2)	0.044 (2)	−0.0068 (17)	0.0064 (17)	−0.028 (2)
O5	0.155 (5)	0.196 (6)	0.125 (5)	−0.039 (5)	−0.060 (4)	−0.013 (4)
C77	0.108 (5)	0.143 (6)	0.083 (5)	−0.013 (5)	0.008 (4)	0.021 (4)
C78	0.065 (4)	0.115 (5)	0.089 (4)	−0.018 (3)	−0.033 (3)	−0.006 (4)
C79	0.137 (6)	0.137 (5)	0.086 (5)	−0.092 (5)	−0.049 (4)	0.042 (4)
C80	0.072 (4)	0.155 (6)	0.084 (4)	0.024 (4)	−0.009 (3)	−0.008 (4)
O6	0.050 (4)	0.061 (4)	0.053 (4)	0.002 (3)	0.003 (3)	−0.015 (3)
C81	0.098 (8)	0.118 (7)	0.087 (7)	−0.027 (6)	−0.021 (6)	0.013 (5)
C82	0.158 (11)	0.185 (9)	0.138 (9)	−0.064 (8)	−0.062 (8)	−0.021 (7)
C83	0.101 (8)	0.146 (8)	0.134 (8)	−0.060 (7)	−0.031 (7)	−0.044 (6)
C84	0.064 (6)	0.074 (5)	0.085 (6)	−0.015 (5)	0.006 (5)	−0.019 (5)
N17	0.0352 (18)	0.0389 (19)	0.0436 (19)	−0.0110 (15)	−0.0109 (15)	−0.0113 (15)
N18	0.0371 (18)	0.0350 (18)	0.051 (2)	−0.0074 (15)	−0.0163 (16)	−0.0121 (16)
C85	0.037 (2)	0.040 (2)	0.050 (3)	−0.0112 (18)	−0.0079 (19)	−0.0130 (19)
C86	0.047 (2)	0.033 (2)	0.061 (3)	−0.0095 (18)	−0.027 (2)	−0.009 (2)
C87	0.045 (2)	0.037 (2)	0.046 (2)	−0.0164 (18)	−0.0099 (19)	−0.0099 (18)
C88	0.051 (3)	0.051 (3)	0.043 (2)	−0.021 (2)	−0.010 (2)	−0.011 (2)

### Geometric parameters (Å, º)

**Table:**

Fe1—N1	1.982 (3)	C35—H35	0.9500
Fe1—N3	1.985 (3)	C36—C37	1.379 (5)
Fe1—N4	1.993 (3)	C36—H36	0.9500
Fe1—N15	1.993 (3)	C37—C38	1.393 (5)
Fe1—N2	2.001 (3)	C37—H37	0.9500
Fe1—N13	2.004 (3)	C39—C40	1.503 (5)
O1—C27	1.225 (5)	C40—C41	1.368 (6)
O2—C39	1.224 (4)	C40—C44	1.387 (5)
O3—C51	1.228 (4)	C41—H41	0.9500
O4—C63	1.228 (4)	C42—C43	1.366 (7)
N1—C4	1.379 (4)	C42—H42	0.9500
N1—C1	1.384 (4)	C43—C44	1.381 (5)
N2—C6	1.374 (4)	C43—H43	0.9500
N2—C9	1.377 (4)	C44—H44	0.9500
N3—C11	1.375 (4)	C45—C46	1.394 (5)
N3—C14	1.376 (4)	C45—C50	1.396 (4)
N4—C16	1.366 (4)	C46—C47	1.385 (5)
N4—C19	1.386 (4)	C46—H46	0.9500
N5—C27	1.348 (5)	C47—C48	1.392 (5)
N5—C26	1.419 (5)	C47—H47	0.9500
N5—H5	0.8800	C48—C49	1.382 (5)
N6—C39	1.349 (4)	C48—H48	0.9500
N6—C38	1.429 (4)	C49—C50	1.383 (5)
N6—H6	0.8800	C49—H49	0.9500
N7—C51	1.348 (5)	C51—C52	1.494 (5)
N7—C50	1.422 (4)	C52—C53	1.382 (5)
N7—H7	0.8800	C52—C56	1.386 (5)
N8—C63	1.348 (5)	C53—C54	1.373 (6)
N8—C62	1.433 (5)	C53—H53	0.9500
N8—H8	0.8800	C54—C55	1.367 (7)
N9—C29	1.336 (5)	C54—H54	0.9500
N9—C30	1.338 (5)	C55—H55	0.9500
N10—C42	1.339 (6)	C56—H56	0.9500
N10—C41	1.352 (5)	C57—C62	1.398 (5)
N11—C56	1.336 (5)	C57—C58	1.402 (5)
N11—C55	1.336 (6)	C58—C59	1.377 (5)
N12—C66	1.344 (7)	C58—H58	0.9500
N12—C65	1.345 (6)	C59—C60	1.372 (6)
N13—C70	1.328 (5)	C59—H59	0.9500
N13—C69	1.358 (5)	C60—C61	1.377 (6)
N14—C71	1.323 (6)	C60—H60	0.9500
N14—C70	1.355 (5)	C61—C62	1.389 (5)
N14—C72	1.458 (6)	C61—H61	0.9500
N15—C73	1.314 (4)	C63—C64	1.497 (5)
N15—C75	1.368 (4)	C64—C65	1.372 (6)
N16—C73	1.345 (4)	C64—C68	1.374 (6)
N16—C74	1.349 (5)	C65—H65	0.9500
N16—C76	1.457 (4)	C66—C67	1.323 (7)
C1—C20	1.392 (5)	C66—H66	0.9500
C1—C2	1.439 (5)	C67—C68	1.373 (7)
C2—C3	1.344 (5)	C67—H67	0.9500
C2—H2	0.9500	C68—H68	0.9500
C3—C4	1.436 (5)	C69—C71	1.357 (6)
C3—H3	0.9500	C69—H69	0.9500
C4—C5	1.400 (5)	C70—H70	0.9500
C5—C6	1.392 (5)	C71—H71	0.9500
C5—C21	1.494 (4)	C72—H72A	0.9800
C6—C7	1.439 (5)	C72—H72B	0.9800
C7—C8	1.344 (5)	C72—H72C	0.9800
C7—H7A	0.9500	C73—H73	0.9500
C8—C9	1.441 (5)	C74—C75	1.356 (5)
C8—H8A	0.9500	C74—H74	0.9500
C9—C10	1.391 (4)	C75—H75	0.9500
C10—C11	1.395 (4)	C76—H76A	0.9800
C10—C33	1.502 (4)	C76—H76B	0.9800
C11—C12	1.437 (4)	C76—H76C	0.9800
C12—C13	1.339 (5)	O5—C80	1.394 (7)
C12—H12	0.9500	O5—C78	1.427 (7)
C13—C14	1.446 (4)	C77—C78	1.498 (8)
C13—H13	0.9500	C77—C79	1.548 (8)
C14—C15	1.389 (4)	C77—H77A	0.9900
C15—C16	1.393 (4)	C77—H77B	0.9900
C15—C45	1.497 (4)	C78—H78A	0.9900
C16—C17	1.444 (4)	C78—H78B	0.9900
C17—C18	1.341 (5)	C79—C80	1.457 (8)
C17—H17	0.9500	C79—H79A	0.9900
C18—C19	1.447 (4)	C79—H79B	0.9900
C18—H18	0.9500	C80—H80A	0.9900
C19—C20	1.390 (5)	C80—H80B	0.9900
C20—C57	1.494 (4)	O6—C84	1.417 (8)
C21—C22	1.386 (5)	O6—C81	1.428 (9)
C21—C26	1.408 (5)	C81—C82	1.552 (10)
C22—C23	1.389 (5)	C81—H81A	0.9900
C22—H22	0.9500	C81—H81B	0.9900
C23—C24	1.384 (6)	C82—C83	1.533 (10)
C23—H23	0.9500	C82—H82A	0.9900
C24—C25	1.387 (6)	C82—H82B	0.9900
C24—H24	0.9500	C83—C84	1.528 (9)
C25—C26	1.390 (5)	C83—H83A	0.9900
C25—H25	0.9500	C83—H83B	0.9900
C27—C28	1.500 (6)	C84—H84A	0.9900
C28—C29	1.392 (5)	C84—H84B	0.9900
C28—C32	1.397 (5)	N17—C86	1.350 (5)
C29—H29	0.9500	N17—C87	1.363 (5)
C30—C31	1.382 (7)	N17—C88	1.456 (5)
C30—H30	0.9500	N18—C86	1.317 (6)
C31—C32	1.377 (7)	N18—C85	1.373 (5)
C31—H31	0.9500	C85—C87	1.377 (6)
C32—H32	0.9500	C85—H85	0.9500
C33—C38	1.390 (4)	C86—H86	0.9500
C33—C34	1.400 (4)	C87—H87	0.9500
C34—C35	1.385 (5)	C88—H88A	0.9800
C34—H34	0.9500	C88—H88B	0.9800
C35—C36	1.372 (5)	C88—H88C	0.9800
			
N1—Fe1—N3	178.48 (11)	N10—C42—H42	118.2
N1—Fe1—N4	89.13 (11)	C43—C42—H42	118.2
N3—Fe1—N4	90.55 (10)	C42—C43—C44	119.2 (4)
N1—Fe1—N15	88.61 (11)	C42—C43—H43	120.4
N3—Fe1—N15	89.91 (10)	C44—C43—H43	120.4
N4—Fe1—N15	89.74 (10)	C43—C44—C40	118.5 (4)
N1—Fe1—N2	90.97 (11)	C43—C44—H44	120.8
N3—Fe1—N2	89.38 (10)	C40—C44—H44	120.8
N4—Fe1—N2	178.69 (11)	C46—C45—C50	118.0 (3)
N15—Fe1—N2	91.57 (11)	C46—C45—C15	119.9 (3)
N1—Fe1—N13	90.73 (11)	C50—C45—C15	122.1 (3)
N3—Fe1—N13	90.75 (11)	C47—C46—C45	121.6 (3)
N4—Fe1—N13	90.23 (11)	C47—C46—H46	119.2
N15—Fe1—N13	179.34 (11)	C45—C46—H46	119.2
N2—Fe1—N13	88.47 (11)	C46—C47—C48	119.5 (3)
C4—N1—C1	105.3 (3)	C46—C47—H47	120.2
C4—N1—Fe1	126.2 (2)	C48—C47—H47	120.2
C1—N1—Fe1	128.4 (2)	C49—C48—C47	119.4 (3)
C6—N2—C9	105.1 (3)	C49—C48—H48	120.3
C6—N2—Fe1	126.5 (2)	C47—C48—H48	120.3
C9—N2—Fe1	128.0 (2)	C48—C49—C50	121.0 (3)
C11—N3—C14	105.2 (2)	C48—C49—H49	119.5
C11—N3—Fe1	127.9 (2)	C50—C49—H49	119.5
C14—N3—Fe1	126.8 (2)	C49—C50—C45	120.5 (3)
C16—N4—C19	105.0 (3)	C49—C50—N7	119.4 (3)
C16—N4—Fe1	127.0 (2)	C45—C50—N7	120.1 (3)
C19—N4—Fe1	128.0 (2)	O3—C51—N7	124.2 (3)
C27—N5—C26	129.9 (3)	O3—C51—C52	120.6 (3)
C27—N5—H5	115.1	N7—C51—C52	115.2 (3)
C26—N5—H5	115.1	C53—C52—C56	118.1 (4)
C39—N6—C38	119.9 (3)	C53—C52—C51	122.7 (3)
C39—N6—H6	120.1	C56—C52—C51	119.1 (3)
C38—N6—H6	120.1	C54—C53—C52	118.7 (4)
C51—N7—C50	122.9 (3)	C54—C53—H53	120.6
C51—N7—H7	118.5	C52—C53—H53	120.6
C50—N7—H7	118.5	C55—C54—C53	119.1 (4)
C63—N8—C62	122.7 (3)	C55—C54—H54	120.4
C63—N8—H8	118.7	C53—C54—H54	120.4
C62—N8—H8	118.7	N11—C55—C54	123.8 (4)
C29—N9—C30	117.0 (4)	N11—C55—H55	118.1
C42—N10—C41	116.4 (4)	C54—C55—H55	118.1
C56—N11—C55	116.6 (4)	N11—C56—C52	123.6 (4)
C66—N12—C65	116.5 (4)	N11—C56—H56	118.2
C70—N13—C69	104.7 (3)	C52—C56—H56	118.2
C70—N13—Fe1	127.5 (3)	C62—C57—C58	117.4 (3)
C69—N13—Fe1	127.7 (3)	C62—C57—C20	124.2 (3)
C71—N14—C70	106.9 (3)	C58—C57—C20	118.4 (3)
C71—N14—C72	126.6 (4)	C59—C58—C57	121.5 (4)
C70—N14—C72	126.4 (5)	C59—C58—H58	119.3
C73—N15—C75	105.1 (3)	C57—C58—H58	119.3
C73—N15—Fe1	128.0 (2)	C60—C59—C58	120.0 (4)
C75—N15—Fe1	126.9 (2)	C60—C59—H59	120.0
C73—N16—C74	107.1 (3)	C58—C59—H59	120.0
C73—N16—C76	127.1 (3)	C59—C60—C61	120.2 (4)
C74—N16—C76	125.8 (3)	C59—C60—H60	119.9
N1—C1—C20	125.2 (3)	C61—C60—H60	119.9
N1—C1—C2	109.7 (3)	C60—C61—C62	120.2 (4)
C20—C1—C2	125.0 (3)	C60—C61—H61	119.9
C3—C2—C1	107.6 (3)	C62—C61—H61	119.9
C3—C2—H2	126.2	C61—C62—C57	120.7 (3)
C1—C2—H2	126.2	C61—C62—N8	119.1 (3)
C2—C3—C4	106.9 (3)	C57—C62—N8	120.1 (3)
C2—C3—H3	126.5	O4—C63—N8	124.0 (3)
C4—C3—H3	126.5	O4—C63—C64	120.1 (3)
N1—C4—C5	125.9 (3)	N8—C63—C64	115.8 (3)
N1—C4—C3	110.5 (3)	C65—C64—C68	117.8 (4)
C5—C4—C3	123.4 (3)	C65—C64—C63	123.1 (3)
C6—C5—C4	123.8 (3)	C68—C64—C63	119.1 (4)
C6—C5—C21	118.4 (3)	N12—C65—C64	123.3 (4)
C4—C5—C21	117.5 (3)	N12—C65—H65	118.3
N2—C6—C5	125.4 (3)	C64—C65—H65	118.3
N2—C6—C7	110.4 (3)	C67—C66—N12	123.5 (5)
C5—C6—C7	124.1 (3)	C67—C66—H66	118.3
C8—C7—C6	107.1 (3)	N12—C66—H66	118.3
C8—C7—H7A	126.5	C66—C67—C68	120.0 (5)
C6—C7—H7A	126.5	C66—C67—H67	120.0
C7—C8—C9	106.8 (3)	C68—C67—H67	120.0
C7—C8—H8A	126.6	C67—C68—C64	118.8 (5)
C9—C8—H8A	126.6	C67—C68—H68	120.6
N2—C9—C10	125.0 (3)	C64—C68—H68	120.6
N2—C9—C8	110.5 (3)	C71—C69—N13	109.7 (4)
C10—C9—C8	124.5 (3)	C71—C69—H69	125.2
C9—C10—C11	123.5 (3)	N13—C69—H69	125.2
C9—C10—C33	119.1 (3)	N13—C70—N14	111.1 (4)
C11—C10—C33	117.2 (3)	N13—C70—H70	124.4
N3—C11—C10	125.9 (3)	N14—C70—H70	124.4
N3—C11—C12	110.2 (3)	N14—C71—C69	107.5 (3)
C10—C11—C12	123.9 (3)	N14—C71—H71	126.2
C13—C12—C11	107.6 (3)	C69—C71—H71	126.2
C13—C12—H12	126.2	N14—C72—H72A	109.5
C11—C12—H12	126.2	N14—C72—H72B	109.5
C12—C13—C14	106.6 (3)	H72A—C72—H72B	109.5
C12—C13—H13	126.7	N14—C72—H72C	109.5
C14—C13—H13	126.7	H72A—C72—H72C	109.5
N3—C14—C15	125.9 (3)	H72B—C72—H72C	109.5
N3—C14—C13	110.4 (3)	N15—C73—N16	111.7 (3)
C15—C14—C13	123.8 (3)	N15—C73—H73	124.2
C14—C15—C16	123.8 (3)	N16—C73—H73	124.2
C14—C15—C45	118.7 (3)	N16—C74—C75	106.5 (3)
C16—C15—C45	117.5 (3)	N16—C74—H74	126.7
N4—C16—C15	125.8 (3)	C75—C74—H74	126.7
N4—C16—C17	110.9 (3)	C74—C75—N15	109.7 (3)
C15—C16—C17	123.3 (3)	C74—C75—H75	125.2
C18—C17—C16	107.0 (3)	N15—C75—H75	125.2
C18—C17—H17	126.5	N16—C76—H76A	109.5
C16—C17—H17	126.5	N16—C76—H76B	109.5
C17—C18—C19	106.8 (3)	H76A—C76—H76B	109.5
C17—C18—H18	126.6	N16—C76—H76C	109.5
C19—C18—H18	126.6	H76A—C76—H76C	109.5
N4—C19—C20	125.4 (3)	H76B—C76—H76C	109.5
N4—C19—C18	110.2 (3)	C80—O5—C78	109.7 (7)
C20—C19—C18	124.3 (3)	C78—C77—C79	101.3 (7)
C19—C20—C1	123.3 (3)	C78—C77—H77A	111.5
C19—C20—C57	119.0 (3)	C79—C77—H77A	111.5
C1—C20—C57	117.6 (3)	C78—C77—H77B	111.5
C22—C21—C26	118.5 (3)	C79—C77—H77B	111.5
C22—C21—C5	121.9 (3)	H77A—C77—H77B	109.3
C26—C21—C5	119.5 (3)	O5—C78—C77	107.3 (7)
C21—C22—C23	121.5 (4)	O5—C78—H78A	110.3
C21—C22—H22	119.3	C77—C78—H78A	110.3
C23—C22—H22	119.3	O5—C78—H78B	110.3
C24—C23—C22	119.0 (4)	C77—C78—H78B	110.3
C24—C23—H23	120.5	H78A—C78—H78B	108.5
C22—C23—H23	120.5	C80—C79—C77	102.5 (8)
C23—C24—C25	121.1 (4)	C80—C79—H79A	111.3
C23—C24—H24	119.5	C77—C79—H79A	111.3
C25—C24—H24	119.5	C80—C79—H79B	111.3
C24—C25—C26	119.5 (4)	C77—C79—H79B	111.3
C24—C25—H25	120.3	H79A—C79—H79B	109.2
C26—C25—H25	120.3	O5—C80—C79	107.1 (7)
C25—C26—C21	120.4 (3)	O5—C80—H80A	110.3
C25—C26—N5	122.6 (3)	C79—C80—H80A	110.3
C21—C26—N5	117.0 (3)	O5—C80—H80B	110.3
O1—C27—N5	123.6 (4)	C79—C80—H80B	110.3
O1—C27—C28	120.6 (4)	H80A—C80—H80B	108.5
N5—C27—C28	115.7 (3)	C84—O6—C81	113.4 (10)
C29—C28—C32	117.3 (4)	O6—C81—C82	99.7 (11)
C29—C28—C27	124.8 (3)	O6—C81—H81A	111.8
C32—C28—C27	117.8 (4)	C82—C81—H81A	111.8
N9—C29—C28	124.3 (4)	O6—C81—H81B	111.8
N9—C29—H29	117.9	C82—C81—H81B	111.8
C28—C29—H29	117.9	H81A—C81—H81B	109.6
N9—C30—C31	123.2 (4)	C83—C82—C81	113.6 (13)
N9—C30—H30	118.4	C83—C82—H82A	108.9
C31—C30—H30	118.4	C81—C82—H82A	108.9
C32—C31—C30	119.3 (4)	C83—C82—H82B	108.9
C32—C31—H31	120.4	C81—C82—H82B	108.9
C30—C31—H31	120.4	H82A—C82—H82B	107.7
C31—C32—C28	118.9 (4)	C84—C83—C82	96.6 (13)
C31—C32—H32	120.6	C84—C83—H83A	112.4
C28—C32—H32	120.6	C82—C83—H83A	112.4
C38—C33—C34	117.5 (3)	C84—C83—H83B	112.4
C38—C33—C10	124.6 (3)	C82—C83—H83B	112.4
C34—C33—C10	117.9 (3)	H83A—C83—H83B	110.0
C35—C34—C33	121.7 (3)	O6—C84—C83	109.7 (10)
C35—C34—H34	119.1	O6—C84—H84A	109.7
C33—C34—H34	119.1	C83—C84—H84A	109.7
C36—C35—C34	119.8 (3)	O6—C84—H84B	109.7
C36—C35—H35	120.1	C83—C84—H84B	109.7
C34—C35—H35	120.1	H84A—C84—H84B	108.2
C35—C36—C37	119.7 (3)	C86—N17—C87	106.5 (4)
C35—C36—H36	120.1	C86—N17—C88	125.6 (4)
C37—C36—H36	120.1	C87—N17—C88	127.9 (3)
C36—C37—C38	120.7 (3)	C86—N18—C85	104.8 (3)
C36—C37—H37	119.6	N18—C85—C87	109.6 (4)
C38—C37—H37	119.6	N18—C85—H85	125.2
C33—C38—C37	120.5 (3)	C87—C85—H85	125.2
C33—C38—N6	121.5 (3)	N18—C86—N17	112.7 (4)
C37—C38—N6	117.9 (3)	N18—C86—H86	123.6
O2—C39—N6	123.6 (3)	N17—C86—H86	123.6
O2—C39—C40	120.5 (3)	N17—C87—C85	106.3 (4)
N6—C39—C40	115.9 (3)	N17—C87—H87	126.9
C41—C40—C44	118.5 (3)	C85—C87—H87	126.9
C41—C40—C39	123.0 (3)	N17—C88—H88A	109.5
C44—C40—C39	118.5 (3)	N17—C88—H88B	109.5
N10—C41—C40	123.8 (4)	H88A—C88—H88B	109.5
N10—C41—H41	118.1	N17—C88—H88C	109.5
C40—C41—H41	118.1	H88A—C88—H88C	109.5
N10—C42—C43	123.7 (4)	H88B—C88—H88C	109.5
			
C4—N1—C1—C20	175.1 (3)	C34—C35—C36—C37	−0.3 (6)
Fe1—N1—C1—C20	−8.4 (5)	C35—C36—C37—C38	−1.0 (6)
C4—N1—C1—C2	−1.7 (4)	C34—C33—C38—C37	−1.5 (5)
Fe1—N1—C1—C2	174.8 (2)	C10—C33—C38—C37	178.4 (3)
N1—C1—C2—C3	0.4 (4)	C34—C33—C38—N6	176.5 (3)
C20—C1—C2—C3	−176.5 (3)	C10—C33—C38—N6	−3.6 (5)
C1—C2—C3—C4	1.1 (4)	C36—C37—C38—C33	2.0 (5)
C1—N1—C4—C5	−172.4 (3)	C36—C37—C38—N6	−176.1 (3)
Fe1—N1—C4—C5	11.0 (5)	C39—N6—C38—C33	−92.4 (4)
C1—N1—C4—C3	2.4 (4)	C39—N6—C38—C37	85.7 (4)
Fe1—N1—C4—C3	−174.2 (2)	C38—N6—C39—O2	4.1 (5)
C2—C3—C4—N1	−2.3 (4)	C38—N6—C39—C40	−175.1 (3)
C2—C3—C4—C5	172.7 (3)	O2—C39—C40—C41	146.9 (4)
N1—C4—C5—C6	−9.3 (5)	N6—C39—C40—C41	−33.8 (5)
C3—C4—C5—C6	176.5 (3)	O2—C39—C40—C44	−29.8 (5)
N1—C4—C5—C21	164.0 (3)	N6—C39—C40—C44	149.4 (3)
C3—C4—C5—C21	−10.1 (5)	C42—N10—C41—C40	−0.5 (7)
C9—N2—C6—C5	−176.7 (3)	C44—C40—C41—N10	0.5 (6)
Fe1—N2—C6—C5	9.9 (5)	C39—C40—C41—N10	−176.3 (4)
C9—N2—C6—C7	2.5 (4)	C41—N10—C42—C43	0.3 (7)
Fe1—N2—C6—C7	−170.9 (2)	N10—C42—C43—C44	−0.1 (7)
C4—C5—C6—N2	−1.8 (5)	C42—C43—C44—C40	0.0 (6)
C21—C5—C6—N2	−175.1 (3)	C41—C40—C44—C43	−0.2 (6)
C4—C5—C6—C7	179.1 (3)	C39—C40—C44—C43	176.7 (3)
C21—C5—C6—C7	5.8 (5)	C14—C15—C45—C46	110.8 (3)
N2—C6—C7—C8	−3.0 (4)	C16—C15—C45—C46	−68.4 (4)
C5—C6—C7—C8	176.2 (3)	C14—C15—C45—C50	−69.1 (4)
C6—C7—C8—C9	2.2 (4)	C16—C15—C45—C50	111.7 (3)
C6—N2—C9—C10	179.4 (3)	C50—C45—C46—C47	−0.7 (5)
Fe1—N2—C9—C10	−7.3 (5)	C15—C45—C46—C47	179.4 (3)
C6—N2—C9—C8	−1.2 (4)	C45—C46—C47—C48	0.7 (5)
Fe1—N2—C9—C8	172.1 (2)	C46—C47—C48—C49	−0.4 (6)
C7—C8—C9—N2	−0.7 (4)	C47—C48—C49—C50	0.1 (6)
C7—C8—C9—C10	178.8 (3)	C48—C49—C50—C45	−0.1 (5)
N2—C9—C10—C11	6.5 (5)	C48—C49—C50—N7	177.7 (3)
C8—C9—C10—C11	−172.8 (3)	C46—C45—C50—C49	0.4 (5)
N2—C9—C10—C33	−167.5 (3)	C15—C45—C50—C49	−179.7 (3)
C8—C9—C10—C33	13.2 (5)	C46—C45—C50—N7	−177.4 (3)
C14—N3—C11—C10	−179.7 (3)	C15—C45—C50—N7	2.5 (5)
Fe1—N3—C11—C10	−2.8 (4)	C51—N7—C50—C49	117.2 (4)
C14—N3—C11—C12	1.1 (3)	C51—N7—C50—C45	−64.9 (4)
Fe1—N3—C11—C12	178.1 (2)	C50—N7—C51—O3	−9.8 (5)
C9—C10—C11—N3	−1.3 (5)	C50—N7—C51—C52	171.3 (3)
C33—C10—C11—N3	172.8 (3)	O3—C51—C52—C53	143.9 (4)
C9—C10—C11—C12	177.7 (3)	N7—C51—C52—C53	−37.0 (5)
C33—C10—C11—C12	−8.2 (4)	O3—C51—C52—C56	−33.3 (5)
N3—C11—C12—C13	−1.4 (4)	N7—C51—C52—C56	145.7 (3)
C10—C11—C12—C13	179.4 (3)	C56—C52—C53—C54	−1.2 (6)
C11—C12—C13—C14	1.1 (4)	C51—C52—C53—C54	−178.4 (4)
C11—N3—C14—C15	178.5 (3)	C52—C53—C54—C55	0.3 (7)
Fe1—N3—C14—C15	1.5 (4)	C56—N11—C55—C54	−0.3 (7)
C11—N3—C14—C13	−0.5 (3)	C53—C54—C55—N11	0.6 (7)
Fe1—N3—C14—C13	−177.4 (2)	C55—N11—C56—C52	−0.7 (7)
C12—C13—C14—N3	−0.4 (4)	C53—C52—C56—N11	1.5 (6)
C12—C13—C14—C15	−179.4 (3)	C51—C52—C56—N11	178.8 (4)
N3—C14—C15—C16	2.6 (5)	C19—C20—C57—C62	−66.4 (4)
C13—C14—C15—C16	−178.5 (3)	C1—C20—C57—C62	117.7 (4)
N3—C14—C15—C45	−176.5 (3)	C19—C20—C57—C58	115.2 (4)
C13—C14—C15—C45	2.3 (4)	C1—C20—C57—C58	−60.7 (4)
C19—N4—C16—C15	179.9 (3)	C62—C57—C58—C59	−1.7 (6)
Fe1—N4—C16—C15	0.9 (4)	C20—C57—C58—C59	176.8 (4)
C19—N4—C16—C17	1.6 (3)	C57—C58—C59—C60	1.0 (7)
Fe1—N4—C16—C17	−177.5 (2)	C58—C59—C60—C61	0.0 (8)
C14—C15—C16—N4	−3.9 (5)	C59—C60—C61—C62	−0.3 (7)
C45—C15—C16—N4	175.3 (3)	C60—C61—C62—C57	−0.4 (6)
C14—C15—C16—C17	174.2 (3)	C60—C61—C62—N8	−177.6 (4)
C45—C15—C16—C17	−6.6 (4)	C58—C57—C62—C61	1.3 (5)
N4—C16—C17—C18	−1.0 (4)	C20—C57—C62—C61	−177.0 (3)
C15—C16—C17—C18	−179.4 (3)	C58—C57—C62—N8	178.5 (3)
C16—C17—C18—C19	−0.1 (4)	C20—C57—C62—N8	0.1 (5)
C16—N4—C19—C20	174.1 (3)	C63—N8—C62—C61	−51.0 (5)
Fe1—N4—C19—C20	−6.9 (4)	C63—N8—C62—C57	131.8 (4)
C16—N4—C19—C18	−1.6 (3)	C62—N8—C63—O4	−4.4 (5)
Fe1—N4—C19—C18	177.4 (2)	C62—N8—C63—C64	174.8 (3)
C17—C18—C19—N4	1.1 (4)	O4—C63—C64—C65	−135.2 (5)
C17—C18—C19—C20	−174.7 (3)	N8—C63—C64—C65	45.6 (5)
N4—C19—C20—C1	0.6 (5)	O4—C63—C64—C68	44.9 (6)
C18—C19—C20—C1	175.7 (3)	N8—C63—C64—C68	−134.3 (4)
N4—C19—C20—C57	−175.1 (3)	C66—N12—C65—C64	−0.6 (9)
C18—C19—C20—C57	0.0 (5)	C68—C64—C65—N12	−0.4 (8)
N1—C1—C20—C19	7.3 (5)	C63—C64—C65—N12	179.7 (5)
C2—C1—C20—C19	−176.4 (3)	C65—N12—C66—C67	2.1 (10)
N1—C1—C20—C57	−177.0 (3)	N12—C66—C67—C68	−2.5 (10)
C2—C1—C20—C57	−0.6 (5)	C66—C67—C68—C64	1.4 (9)
C6—C5—C21—C22	−72.0 (5)	C65—C64—C68—C67	0.0 (8)
C4—C5—C21—C22	114.3 (4)	C63—C64—C68—C67	179.9 (5)
C6—C5—C21—C26	106.3 (4)	C70—N13—C69—C71	1.0 (5)
C4—C5—C21—C26	−67.4 (4)	Fe1—N13—C69—C71	176.8 (3)
C26—C21—C22—C23	−0.7 (6)	C69—N13—C70—N14	−1.2 (5)
C5—C21—C22—C23	177.7 (4)	Fe1—N13—C70—N14	−177.0 (3)
C21—C22—C23—C24	0.3 (6)	C71—N14—C70—N13	1.0 (5)
C22—C23—C24—C25	−0.5 (7)	C72—N14—C70—N13	177.5 (4)
C23—C24—C25—C26	1.1 (7)	C70—N14—C71—C69	−0.3 (5)
C24—C25—C26—C21	−1.5 (6)	C72—N14—C71—C69	−176.8 (5)
C24—C25—C26—N5	178.0 (4)	N13—C69—C71—N14	−0.4 (5)
C22—C21—C26—C25	1.3 (6)	C75—N15—C73—N16	0.0 (4)
C5—C21—C26—C25	−177.1 (4)	Fe1—N15—C73—N16	−179.7 (2)
C22—C21—C26—N5	−178.2 (3)	C74—N16—C73—N15	0.0 (4)
C5—C21—C26—N5	3.4 (5)	C76—N16—C73—N15	177.6 (3)
C27—N5—C26—C25	−4.4 (7)	C73—N16—C74—C75	0.1 (5)
C27—N5—C26—C21	175.1 (4)	C76—N16—C74—C75	−177.6 (4)
C26—N5—C27—O1	12.7 (7)	N16—C74—C75—N15	−0.1 (5)
C26—N5—C27—C28	−166.9 (4)	C73—N15—C75—C74	0.1 (4)
O1—C27—C28—C29	−158.4 (4)	Fe1—N15—C75—C74	179.8 (3)
N5—C27—C28—C29	21.3 (6)	C80—O5—C78—C77	−1.9 (9)
O1—C27—C28—C32	17.6 (6)	C79—C77—C78—O5	21.6 (9)
N5—C27—C28—C32	−162.8 (4)	C78—C77—C79—C80	−32.8 (8)
C30—N9—C29—C28	0.8 (6)	C78—O5—C80—C79	−20.6 (10)
C32—C28—C29—N9	−1.0 (6)	C77—C79—C80—O5	33.4 (9)
C27—C28—C29—N9	175.0 (4)	C84—O6—C81—C82	−12.7 (16)
C29—N9—C30—C31	0.2 (7)	O6—C81—C82—C83	−5 (2)
N9—C30—C31—C32	−0.8 (9)	C81—C82—C83—C84	18 (2)
C30—C31—C32—C28	0.6 (8)	C81—O6—C84—C83	26.4 (15)
C29—C28—C32—C31	0.3 (7)	C82—C83—C84—O6	−25.4 (16)
C27—C28—C32—C31	−176.0 (4)	C86—N18—C85—C87	−0.8 (5)
C9—C10—C33—C38	−72.8 (4)	C85—N18—C86—N17	1.1 (5)
C11—C10—C33—C38	112.8 (4)	C87—N17—C86—N18	−1.1 (5)
C9—C10—C33—C34	107.1 (3)	C88—N17—C86—N18	180.0 (4)
C11—C10—C33—C34	−67.3 (4)	C86—N17—C87—C85	0.5 (5)
C38—C33—C34—C35	0.1 (5)	C88—N17—C87—C85	179.5 (4)
C10—C33—C34—C35	−179.8 (3)	N18—C85—C87—N17	0.2 (5)
C33—C34—C35—C36	0.8 (5)		

### Hydrogen-bond geometry (Å, º)

**Table:**

*D*—H···*A*	*D*—H	H···*A*	*D*···*A*	*D*—H···*A*
N6—H6···O4^i^	0.88	2.18	2.948 (4)	145
N8—H8···N9	0.88	2.19	3.018 (5)	156

Symmetry code: (i) *x*, *y*+1, *z*.
